# (4*RS*)-Methyl 4-cyano-4-cyclo­hexyl-4-phenyl­butano­ate

**DOI:** 10.1107/S1600536812021320

**Published:** 2012-05-26

**Authors:** Gui-Sheng Sun, Yu-Lai Hu, Dan-Feng Huang, Chang-Ming Xu

**Affiliations:** a College of Chemistry and Chemical Engineering, Northwest Normal University, Lanzhou, Gansu Province 730070, People’s Republic of China

## Abstract

In the crystal structure of the title compound, C_18_H_23_NO_2_, there are only van der Waals inter­actions present. The cyclo­hexyl ring has a chair conformation. The longer axes of the displacement parameters of the non-H atoms forming the ethyl­methyl­carboxyl­ate skeleton are perpendicular to the plane through the non-H atoms of this skeleton.

## Related literature

For general background to pharmaceutical applications of methyl 4-cyano-4-cyclo­hexyl-4-phenyl­butano­ates, see: Hartmann & Batzl (1986 ▶), Hartmann *et al.* (1992 ▶); Fadel & Garcia-Argote (1996 ▶).
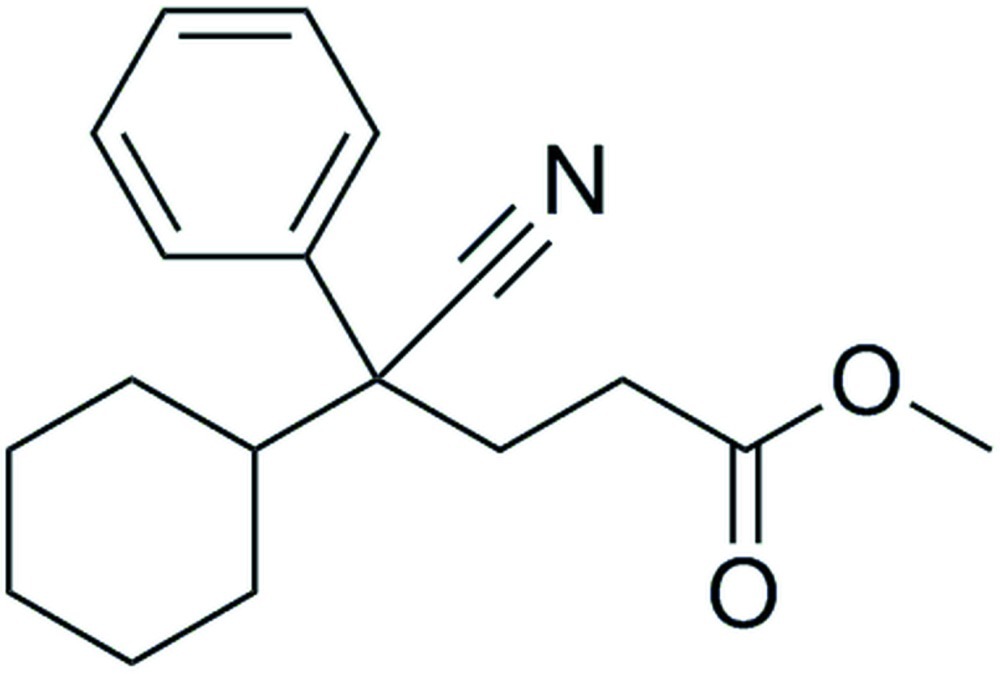


## Experimental

### 

#### Crystal data

C_18_H_23_NO_2_
*M* *_r_* = 285.37
Monoclinic, 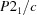

*a* = 8.877 (6) Å
*b* = 9.120 (6) Å
*c* = 20.690 (14) Å
β = 95.769 (6)°
*V* = 1666.5 (19) Å^3^
*Z* = 4
Mo *K*α radiation
μ = 0.07 mm^−1^
*T* = 296 K
0.23 × 0.21 × 0.19 mm

#### Data collection

Bruker APEXII CCD diffractometer
Absorption correction: multi-scan (*SADABS*; Sheldrick, 2008*a* ▶) *T*_min_ = 0.983, *T*_max_ = 0.986
13440 measured reflections
3816 independent reflections
2026 reflections with *I* > 2σ(*I*)
*R*_int_ = 0.041

#### Refinement

*R*[*F* ^2^ > 2σ(*F* ^2^)] = 0.052
*wR*(*F* ^2^) = 0.153
*S* = 1.06
3816 reflections
192 parameters
H-atom parameters constrained
Δρ_max_ = 0.24 e Å^−3^
Δρ_min_ = −0.20 e Å^−3^

### 

Data collection: *APEX2* (Bruker, 2008 ▶); cell refinement: *SAINT* (Bruker, 2008 ▶); data reduction: *SAINT*; program(s) used to solve structure: *SHELXS97* (Sheldrick, 2008*b* ▶); program(s) used to refine structure: *SHELXL97* (Sheldrick, 2008*b* ▶); molecular graphics: *SHELXTL* (Sheldrick, 2008*b* ▶); software used to prepare material for publication: *SHELXTL*.

## Supplementary Material

Crystal structure: contains datablock(s) I, global. DOI: 10.1107/S1600536812021320/fb2251sup1.cif

Structure factors: contains datablock(s) I. DOI: 10.1107/S1600536812021320/fb2251Isup2.hkl

Supplementary material file. DOI: 10.1107/S1600536812021320/fb2251Isup3.cml

Additional supplementary materials:  crystallographic information; 3D view; checkCIF report

## Figures and Tables

**Fig. 1.**
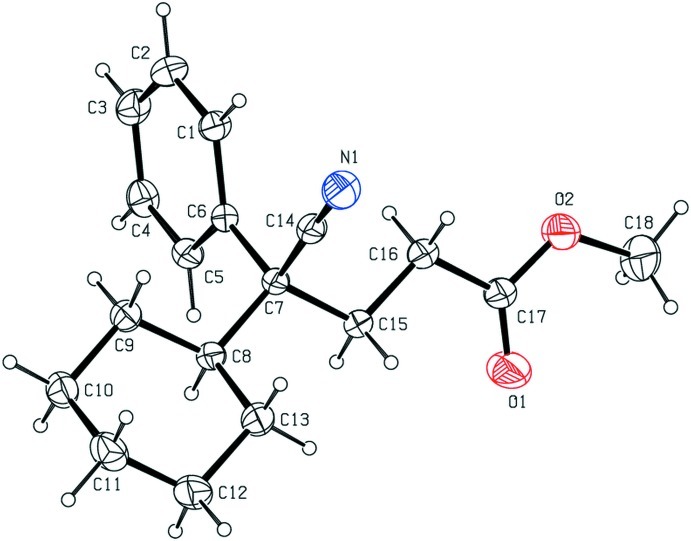
The title molecule with the atom labels and the displacement ellipsoids shown at the 30% probability level.

**Fig. 2.**
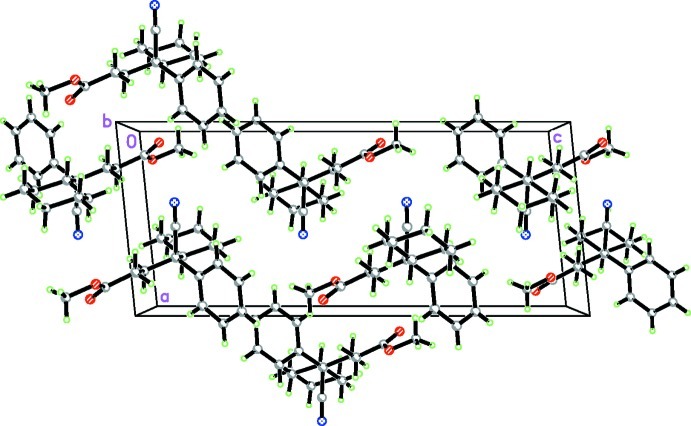
A packing diagram, viewed approximately along the b axis. Applied colourse for the atomic species: C - grey, H - green, N - blue; O - red.

## supplementary crystallographic information

### Comment

Methyl 4-cyano-4-cyclohexyl-4-phenylbutanoates have attracted widespread attention as a pharmaceutical material to synthesis of 3-(4-aminophenyl)-3-cyclohexylpiperidine-2,6-dione (Fadel & Garcia-Argote, 1996). A variety of 3-(4-aminophenyl)-3-cyclohexylpiperidine-2,6-dione derivatives have been screened as inhibitors of human placental aromatase and treatment of estrogen-dependent diseases (Hartmann & Batzl, 1986, and Hartmann *et al.*, 1992).

The title molecule is shown in Fig. 1. The cyclohexane ring adopts a chair conformation. There are no weak hydrogen bonds and no significant intermolecular π–π electron interactions in the structure. The longer axes of the displacement parameters of the non-hydrogen atoms forming the ethylmethylcarboxylate skeleton are perpendicular to the plane through the non-hydrogen atoms through this skeleton. The displacement parameters of these atoms are rather elongated, possibly due to weak intermolecular interactions between the molecules which may enable more intense thermal agitation of the molecules in the structure.

Fig. 2 shows the packing of the molecules in the title structure.

### Experimental

Powdered potassium carbonate (6.9 g, 0.05 mol) and tetrabutylammonium bromide (0.8 g, 0.0025 mol) were added to a solution of 2-cyclohexyl-2-phenylacetonitrile (9.95 g, 0.05 mol) in toluene (35 ml). Methyl acrylate (5.16 g, 0.06 mol) was slowly added in the mixture at 110 °C. After refluxing for 7h, the mixture was cooled and water was added, extracted with ethyl acetate (3 × 50 ml), combined organic solutions, washed with water (3 × 50 ml), and dried over MgSO_4_. The volatiles were removed in vacuo and the crude product was purified by column chromatography over silica gel with ethyl acetate/petroleum ether (1:20 *v*/*v*). The title compound was isolated in 96% yield as a white solid. Colourless transparent block-like crystals with approx. dimensions 0.2 × 0.4 × 0.5 mm were obtained by slow evaporation of ethyl acetate/petroleum ether (1:20 *v*/*v*).

### Refinement

All the H atoms were located in the difference electron density map. Nevertheless, the H atoms were constrained and refined in the riding motion approximation: C_aryl_—H = 0.93, C_methine_—H = 0.98, C_methylene_—H = 0.97, C_methyl_—H = 0.96 Å. *U*_iso_(H_aryl/methane/methylene_) = 1.2 × *U*_eq_(C_carrier_) and *U*_iso_(H_methyl_) = 1.5 × *U*_eq_ (C_carrier_).

### Crystal data

**Table:**

C_18_H_23_NO_2_	*F*(000) = 616
*M**_r_* = 285.37	*D*_x_ = 1.137 Mg m^−^^3^
Monoclinic, *P*2_1_/*c*	Mo *K*α radiation, λ = 0.71073 Å
Hall symbol: -P 2ybc	Cell parameters from 2935 reflections
*a* = 8.877 (6) Å	θ = 2.4–24.9°
*b* = 9.120 (6) Å	µ = 0.07 mm^−^^1^
*c* = 20.690 (14) Å	*T* = 296 K
β = 95.769 (6)°	Block, colourless
*V* = 1666.5 (19) Å^3^	0.23 × 0.21 × 0.19 mm
*Z* = 4	

### Data collection

**Table:**

Bruker APEXII CCD diffractometer	3816 independent reflections
Radiation source: fine-focus sealed tube	2026 reflections with *I* > 2σ(*I*)
Graphite monochromator	*R*_int_ = 0.041
φ and ω scans	θ_max_ = 27.5°, θ_min_ = 2.4°
Absorption correction: multi-scan (*SADABS*; Sheldrick, 2008*a*)	*h* = −11→11
*T*_min_ = 0.983, *T*_max_ = 0.986	*k* = −10→11
13440 measured reflections	*l* = −26→26

### Refinement

**Table:**

Refinement on *F*^2^	Secondary atom site location: difference Fourier map
Least-squares matrix: full	Hydrogen site location: difference Fourier map
*R*[*F*^2^ > 2σ(*F*^2^)] = 0.052	H-atom parameters constrained
*wR*(*F*^2^) = 0.153	*w* = 1/[σ^2^(*F*_o_^2^) + (0.0382*P*)^2^ + 0.7938*P*] where *P* = (*F*_o_^2^ + 2*F*_c_^2^)/3
*S* = 1.06	(Δ/σ)_max_ < 0.001
3816 reflections	Δρ_max_ = 0.24 e Å^−^^3^
192 parameters	Δρ_min_ = −0.20 e Å^−^^3^
0 restraints	Extinction correction: *SHELXL97* (Sheldrick, 2008*b*), Fc^*^=kFc[1+0.001xFc^2^λ^3^/sin(2θ)]^-1/4^
91 constraints	Extinction coefficient: 0.0181 (18)
Primary atom site location: structure-invariant direct methods	

### Special details

**Table:**

Geometry. All e.s.d.'s (except the e.s.d. in the dihedral angle between two l.s. planes) are estimated using the full covariance matrix. The cell e.s.d.'s are taken into account individually in the estimation of e.s.d.'s in distances, angles and torsion angles; correlations between e.s.d.'s in cell parameters are only used when they are defined by crystal symmetry. An approximate (isotropic) treatment of cell e.s.d.'s is used for estimating e.s.d.'s involving l.s. planes.
Refinement. Refinement of *F*^2^ against ALL reflections. The weighted *R*-factor *wR* and goodness of fit *S* are based on *F*^2^, conventional *R*-factors *R* are based on *F*, with *F* set to zero for negative *F*^2^. The threshold expression of *F*^2^ > σ(*F*^2^) is used only for calculating *R*-factors(gt) *etc*. and is not relevant to the choice of reflections for refinement. *R*-factors based on *F*^2^ are statistically about twice as large as those based on *F*, and *R*- factors based on ALL data will be even larger.

### Fractional atomic coordinates and isotropic or equivalent isotropic displacement parameters (Å^2^)

**Table:**

	*x*	*y*	*z*	*U*_iso_*/*U*_eq_	
C1	0.2736 (3)	0.2805 (2)	0.70020 (10)	0.0531 (6)	
H1	0.3761	0.2581	0.7018	0.064*	
C2	0.1862 (3)	0.2158 (3)	0.74406 (11)	0.0652 (7)	
H2	0.2305	0.1505	0.7748	0.078*	
C3	0.0358 (3)	0.2470 (3)	0.74258 (12)	0.0666 (7)	
H3	−0.0221	0.2035	0.7723	0.080*	
C4	−0.0298 (3)	0.3430 (3)	0.69695 (12)	0.0613 (6)	
H4	−0.1326	0.3639	0.6956	0.074*	
C5	0.0566 (2)	0.4086 (3)	0.65311 (11)	0.0537 (6)	
H5	0.0116	0.4740	0.6225	0.064*	
C6	0.2101 (2)	0.3778 (2)	0.65421 (9)	0.0431 (5)	
C7	0.3020 (2)	0.4512 (2)	0.60391 (9)	0.0438 (5)	
C8	0.3038 (2)	0.6215 (2)	0.61212 (10)	0.0478 (5)	
H8	0.1991	0.6555	0.6031	0.057*	
C9	0.3583 (3)	0.6683 (2)	0.68142 (10)	0.0539 (6)	
H9A	0.2947	0.6233	0.7113	0.065*	
H9B	0.4611	0.6339	0.6924	0.065*	
C10	0.3539 (3)	0.8345 (3)	0.68922 (12)	0.0664 (7)	
H10A	0.2500	0.8681	0.6819	0.080*	
H10B	0.3924	0.8605	0.7333	0.080*	
C11	0.4475 (3)	0.9097 (3)	0.64200 (15)	0.0814 (8)	
H11A	0.4389	1.0152	0.6465	0.098*	
H11B	0.5532	0.8832	0.6517	0.098*	
C12	0.3937 (4)	0.8653 (3)	0.57336 (14)	0.0828 (9)	
H12A	0.4576	0.9109	0.5437	0.099*	
H12B	0.2911	0.9004	0.5625	0.099*	
C13	0.3972 (3)	0.6996 (3)	0.56456 (12)	0.0712 (8)	
H13A	0.5011	0.6656	0.5712	0.085*	
H13B	0.3576	0.6751	0.5205	0.085*	
C14	0.4603 (3)	0.3992 (3)	0.61248 (10)	0.0525 (6)	
C15	0.2354 (3)	0.4061 (2)	0.53446 (10)	0.0519 (6)	
H15A	0.1331	0.4440	0.5265	0.062*	
H15B	0.2954	0.4507	0.5031	0.062*	
C16	0.2321 (3)	0.2420 (3)	0.52422 (11)	0.0638 (7)	
H16A	0.3351	0.2051	0.5299	0.077*	
H16B	0.1773	0.1972	0.5574	0.077*	
C17	0.1604 (3)	0.1957 (3)	0.45895 (11)	0.0600 (6)	
C18	0.1176 (4)	−0.0077 (4)	0.38785 (14)	0.1048 (11)	
H18A	0.1542	0.0453	0.3525	0.157*	
H18B	0.0091	−0.0021	0.3846	0.157*	
H18C	0.1481	−0.1085	0.3860	0.157*	
N1	0.5830 (3)	0.3602 (3)	0.61735 (10)	0.0782 (7)	
O1	0.0970 (3)	0.2726 (2)	0.41959 (11)	0.1196 (9)	
O2	0.1799 (3)	0.0558 (2)	0.44876 (9)	0.0983 (7)	

### Atomic displacement parameters (Å^2^)

**Table:**

	*U*^11^	*U*^22^	*U*^33^	*U*^12^	*U*^13^	*U*^23^
C1	0.0528 (13)	0.0558 (14)	0.0509 (12)	0.0029 (11)	0.0056 (10)	0.0068 (11)
C2	0.0753 (18)	0.0634 (17)	0.0575 (14)	−0.0026 (14)	0.0097 (12)	0.0161 (12)
C3	0.0739 (18)	0.0654 (17)	0.0638 (15)	−0.0129 (14)	0.0233 (13)	0.0035 (13)
C4	0.0519 (14)	0.0630 (16)	0.0714 (16)	−0.0043 (12)	0.0185 (12)	−0.0075 (13)
C5	0.0542 (14)	0.0514 (14)	0.0556 (13)	0.0078 (11)	0.0065 (10)	0.0024 (11)
C6	0.0487 (12)	0.0408 (12)	0.0401 (10)	0.0021 (9)	0.0063 (9)	−0.0015 (9)
C7	0.0475 (12)	0.0452 (13)	0.0393 (10)	0.0096 (10)	0.0066 (9)	0.0034 (9)
C8	0.0526 (13)	0.0461 (13)	0.0456 (11)	0.0047 (10)	0.0086 (9)	0.0050 (10)
C9	0.0567 (14)	0.0526 (14)	0.0534 (13)	−0.0002 (11)	0.0096 (10)	0.0007 (11)
C10	0.0753 (17)	0.0576 (16)	0.0672 (16)	0.0004 (13)	0.0113 (13)	−0.0077 (13)
C11	0.095 (2)	0.0509 (16)	0.101 (2)	−0.0073 (15)	0.0258 (17)	−0.0033 (15)
C12	0.115 (2)	0.0532 (17)	0.085 (2)	−0.0024 (16)	0.0358 (17)	0.0148 (15)
C13	0.101 (2)	0.0556 (16)	0.0617 (15)	−0.0003 (14)	0.0320 (14)	0.0084 (12)
C14	0.0559 (14)	0.0576 (15)	0.0452 (12)	0.0075 (12)	0.0116 (10)	0.0051 (10)
C15	0.0653 (14)	0.0503 (14)	0.0398 (11)	0.0109 (11)	0.0045 (10)	0.0034 (10)
C16	0.0911 (19)	0.0530 (15)	0.0457 (12)	0.0089 (13)	−0.0007 (12)	0.0013 (11)
C17	0.0696 (16)	0.0571 (16)	0.0514 (13)	0.0059 (13)	−0.0031 (11)	0.0007 (12)
C18	0.143 (3)	0.085 (2)	0.081 (2)	−0.026 (2)	−0.0188 (19)	−0.0271 (18)
N1	0.0605 (14)	0.0992 (19)	0.0761 (15)	0.0187 (13)	0.0131 (11)	0.0069 (13)
O1	0.175 (2)	0.0836 (16)	0.0866 (15)	0.0319 (15)	−0.0539 (15)	−0.0107 (12)
O2	0.158 (2)	0.0592 (13)	0.0704 (12)	0.0056 (12)	−0.0256 (12)	−0.0117 (10)

### Geometric parameters (Å, º)

**Table:**

C1—C6	1.379 (3)	C10—H10B	0.9700
C1—C2	1.384 (3)	C11—C12	1.508 (4)
C1—H1	0.9300	C11—H11A	0.9700
C2—C3	1.362 (3)	C11—H11B	0.9700
C2—H2	0.9300	C12—C13	1.523 (4)
C3—C4	1.374 (4)	C12—H12A	0.9700
C3—H3	0.9300	C12—H12B	0.9700
C4—C5	1.382 (3)	C13—H13A	0.9700
C4—H4	0.9300	C13—H13B	0.9700
C5—C6	1.389 (3)	C14—N1	1.140 (3)
C5—H5	0.9300	C15—C16	1.512 (3)
C6—C7	1.538 (3)	C15—H15A	0.9700
C7—C14	1.477 (3)	C15—H15B	0.9700
C7—C15	1.553 (3)	C16—C17	1.495 (3)
C7—C8	1.562 (3)	C16—H16A	0.9700
C8—C13	1.525 (3)	C16—H16B	0.9700
C8—C9	1.528 (3)	C17—O1	1.174 (3)
C8—H8	0.9800	C17—O2	1.308 (3)
C9—C10	1.525 (3)	C18—O2	1.445 (3)
C9—H9A	0.9700	C18—H18A	0.9600
C9—H9B	0.9700	C18—H18B	0.9600
C10—C11	1.510 (4)	C18—H18C	0.9600
C10—H10A	0.9700		
			
C6—C1—C2	120.6 (2)	C12—C11—C10	110.1 (2)
C6—C1—H1	119.7	C12—C11—H11A	109.6
C2—C1—H1	119.7	C10—C11—H11A	109.6
C3—C2—C1	120.6 (2)	C12—C11—H11B	109.6
C3—C2—H2	119.7	C10—C11—H11B	109.6
C1—C2—H2	119.7	H11A—C11—H11B	108.1
C2—C3—C4	119.7 (2)	C11—C12—C13	111.8 (2)
C2—C3—H3	120.1	C11—C12—H12A	109.3
C4—C3—H3	120.1	C13—C12—H12A	109.3
C3—C4—C5	120.1 (2)	C11—C12—H12B	109.3
C3—C4—H4	119.9	C13—C12—H12B	109.3
C5—C4—H4	119.9	H12A—C12—H12B	107.9
C4—C5—C6	120.7 (2)	C12—C13—C8	111.6 (2)
C4—C5—H5	119.7	C12—C13—H13A	109.3
C6—C5—H5	119.7	C8—C13—H13A	109.3
C1—C6—C5	118.2 (2)	C12—C13—H13B	109.3
C1—C6—C7	122.61 (19)	C8—C13—H13B	109.3
C5—C6—C7	119.14 (18)	H13A—C13—H13B	108.0
C14—C7—C6	110.00 (17)	N1—C14—C7	178.1 (2)
C14—C7—C15	107.19 (17)	C16—C15—C7	113.07 (17)
C6—C7—C15	109.40 (18)	C16—C15—H15A	109.0
C14—C7—C8	107.87 (18)	C7—C15—H15A	109.0
C6—C7—C8	111.05 (16)	C16—C15—H15B	109.0
C15—C7—C8	111.26 (16)	C7—C15—H15B	109.0
C13—C8—C9	109.49 (19)	H15A—C15—H15B	107.8
C13—C8—C7	113.23 (18)	C17—C16—C15	113.97 (19)
C9—C8—C7	112.28 (17)	C17—C16—H16A	108.8
C13—C8—H8	107.2	C15—C16—H16A	108.8
C9—C8—H8	107.2	C17—C16—H16B	108.8
C7—C8—H8	107.2	C15—C16—H16B	108.8
C10—C9—C8	111.61 (19)	H16A—C16—H16B	107.7
C10—C9—H9A	109.3	O1—C17—O2	122.2 (2)
C8—C9—H9A	109.3	O1—C17—C16	126.1 (2)
C10—C9—H9B	109.3	O2—C17—C16	111.7 (2)
C8—C9—H9B	109.3	O2—C18—H18A	109.5
H9A—C9—H9B	108.0	O2—C18—H18B	109.5
C11—C10—C9	111.2 (2)	H18A—C18—H18B	109.5
C11—C10—H10A	109.4	O2—C18—H18C	109.5
C9—C10—H10A	109.4	H18A—C18—H18C	109.5
C11—C10—H10B	109.4	H18B—C18—H18C	109.5
C9—C10—H10B	109.4	C17—O2—C18	119.1 (2)
H10A—C10—H10B	108.0		
			
C6—C1—C2—C3	0.0 (4)	C6—C7—C8—C9	53.8 (2)
C1—C2—C3—C4	0.3 (4)	C15—C7—C8—C9	175.90 (17)
C2—C3—C4—C5	−0.5 (4)	C13—C8—C9—C10	55.4 (3)
C3—C4—C5—C6	0.4 (4)	C7—C8—C9—C10	−177.91 (19)
C2—C1—C6—C5	−0.1 (3)	C8—C9—C10—C11	−57.1 (3)
C2—C1—C6—C7	−179.3 (2)	C9—C10—C11—C12	56.5 (3)
C4—C5—C6—C1	−0.1 (3)	C10—C11—C12—C13	−56.4 (3)
C4—C5—C6—C7	179.1 (2)	C11—C12—C13—C8	56.4 (3)
C1—C6—C7—C14	1.1 (3)	C9—C8—C13—C12	−54.9 (3)
C5—C6—C7—C14	−178.02 (19)	C7—C8—C13—C12	179.0 (2)
C1—C6—C7—C15	118.6 (2)	C14—C7—C15—C16	62.0 (2)
C5—C6—C7—C15	−60.5 (2)	C6—C7—C15—C16	−57.2 (2)
C1—C6—C7—C8	−118.2 (2)	C8—C7—C15—C16	179.7 (2)
C5—C6—C7—C8	62.6 (2)	C7—C15—C16—C17	176.7 (2)
C14—C7—C8—C13	57.8 (2)	C15—C16—C17—O1	−7.4 (4)
C6—C7—C8—C13	178.43 (18)	C15—C16—C17—O2	170.7 (2)
C15—C7—C8—C13	−59.5 (3)	O1—C17—O2—C18	−1.8 (5)
C14—C7—C8—C9	−66.8 (2)	C16—C17—O2—C18	180.0 (2)
